# Myocardial remodeling in reperfused myocardial infarction with multiparametric CMR: does diffuse fibrosis occur in remote noninfarcted myocardium?

**DOI:** 10.1186/1532-429X-15-S1-P137

**Published:** 2013-01-30

**Authors:** Jonathan A Pan, Peter M Pollak, David Lopez, Christopher M Kramer, Mark Yeager, Michael Salerno

**Affiliations:** 1 Medicine, University of Virginia, Charlottesville, VA, USA; 2 Biomedical Engineering, University of Virginia, Charlottesville, VA, USA; 3 Molecular Physiology and Biological Physics, University of Virginia, Charlottesville, VA, USA; 4 Radiology, University of Virginia, Charlottesville, VA, USA

## Background

Although mortality from myocardial infarction (MI) has decreased due to improved reperfusion strategies, adverse left ventricular (LV) remodeling after MI is associated with poor long-term prognosis and is thus an important therapeutic target. We developed a Yucatan mini-pig model of reperfused MI using percutaneous techniques for coronary occlusion/reperfusion, and sought to characterize remodeling in both the infarct and remote territories using multiparametric CMR techniques.

## Methods

To create the infarct, a 9.0 x 2.5 mm angioplasty balloon was inflated in the LAD distal to the 2nd diagonal branch for 90 minutes followed by reperfusion. CMR imaging was performed at baseline, 2 days (d) and 30d post-MI. The protocol included SSFP cine, T2 mapping with a T2-prep, T1 mapping using a modified MOLLI technique both pre- and 10 minutes after injection of 0.2 mmol/kg of Gd-DTPA, and delayed enhancement (DE) imaging. LV volumes and function were quantified from the cine images, and infarct size was determined from DE images. The T2s and T1s pre- and post-contrast were measured at baseline and in the infarct and remote territories at 2d and 30d post-MI. Additionally partition coefficient (λ) maps were generated.

## Results

**Figure 1:**
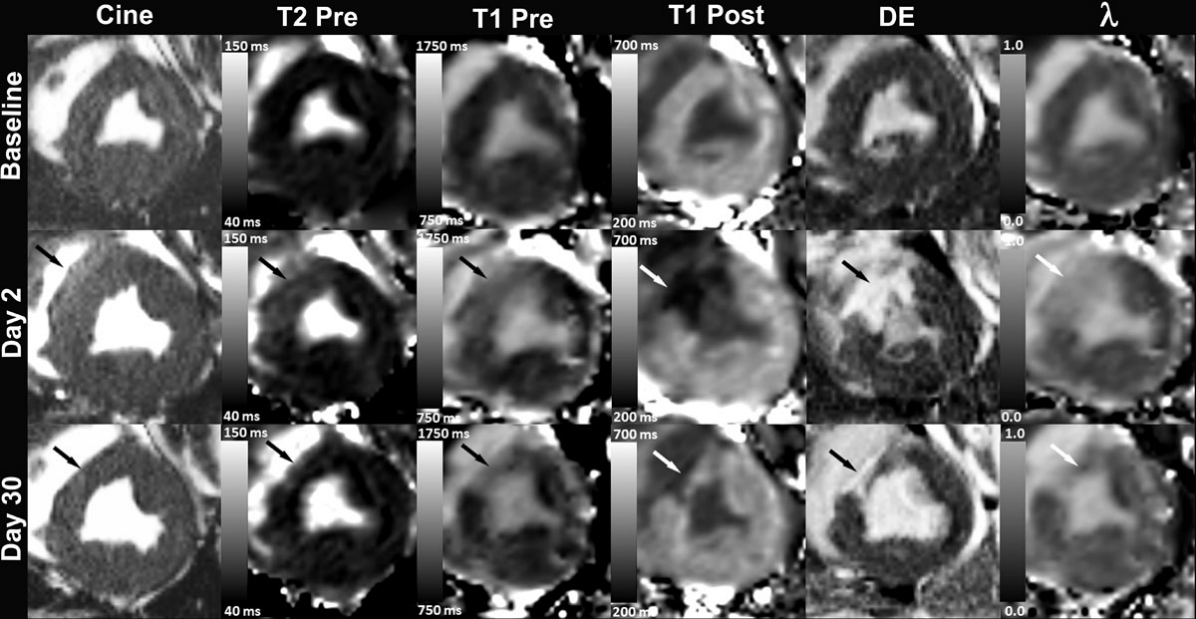
Multiparametric CMR images from one pig at baseline, day 2, and day 30 showing an anteroseptal infarct (arrow). At day 2 there is thickening of the infarct region with prominent edema and nearly transmural DE. At day 30 the infarct region is thinned with evidence of remodeling. (DE = Delayed enhancement; λ = Partition Coefficient)

Eight animals with an infarct size greater than 10% of the LV were analyzed. Figure 1 shows an example case. Multiparametric CMR data is summarized in Table 1. There was an increase in LV volumes and a reduction in EF at one month. The average infarct size at 2d was 22±7%, which decreased to 13±4% by 30d. T2 did not change in the noninfarcted myocardium at any timepoint. In the infarct region, T2 was increased and remained elevated at 30d. Pre-contrast noninfarcted myocardium T1 was increased at 2d, but returned to baseline values by 30d. Similar to T2, the infarct zone pre-contrast T1 was increased and remained elevated at 30d. The λ of noninfarcted myocardium did not change significantly from baseline to 30d post-MI. The infarct region λ increased by more than two-fold at 2d, and remained elevated at one month.

**Table 1:** Summary of multiparametric CMR data at each timepoint.

	Time point post-MI		
	Baseline	Day 2	Day 30

End-diastolic volume (ml)	54.7 ± 13.1	61.0 ± 11.0	70.4 ± 9.6*
End-systolic volume (ml)	22.7 ± 8.4	29.4 ± 8.3	38.6 ± 10.2*
Ejection fraction (%)	59.3 ± 7.4	52.0 ± 9.3	45.6 ± 10.4*
Infarct size (%)	-	21.7 ± 7.3	13.1 ± 4.6†
T2, noninfarcted (ms)	492 ± 44	484 ± 58	501 ± 109
T2, infarct (ms)	-	653 ± 57‡	670 ± 106‡
T1 pre, noninfarcted (ms)	895 ± 52	939 ± 23*	916 ± 26
T1 pre, infarct (ms)	-	1000 ± 54‡	1089 ± 30‡
T1 post, noninfarcted (ms)	486 ± 53	530 ± 61	491 ± 42.0
T1 post, infarct (ms)	-	327 ± 58‡	330 ± 46‡
λ, non-infarcted	0.37 ± 0.02	0.37 ± 0.03	0.39 ± 0.03
λ, infarct	-	0.967 ± 0.25‡	0.881 ± 0.13‡

Data presented as mean ± SD. (λ = Partition Coefficient) * p-value < 0.05 vs. Baseline. † p-value < 0.05 vs. Day 2. ‡ p-value < 0.05 vs. non-infarcted myocardium at Baseline, Day 2 and Day 30.

## Conclusions

Adverse infarct remodeling is readily characterized by multiparametric CMR imaging. In this model T2 and pre-contrast T1 remained elevated in the infarct zone 30d post-MI. Notably, there was no significant acute or late change in λ of the remote noninfarcted myocardium. Thus, there is no evidence of remote fibrosis during LV remodeling. There is, however, a large magnitude change in infarct λ which may be an important parameter for monitoring LV remodeling.

## Funding

AHA 10SDG2650038, NIH K23 HL112910-01, 5T32EB003841, UVA/Astra-Zeneca Research Alliance

